# A taxonomy of indicators for non-communicable diseases: agreement, definition, and contextual description using diabetes as a case study

**DOI:** 10.3389/fpubh.2025.1685731

**Published:** 2025-10-13

**Authors:** Fabrizio Carinci, Tamara Poljičanin, Scott Cunningham, Stephen Fava, Iztok Štotl, Jana Lepiksone, Nicholas Nicholson, Massimo Massi Benedetti, Niek Klazinga

**Affiliations:** ^1^Unicamillus International Medical University, Rome, Italy; ^2^Zagreb County Health Center, Samobor, Croatia; ^3^Clinical Technology Centre, Ninewells Hospital, Dundee, United Kingdom; ^4^Department of Medicine, Mater Dei Hospital of Malta, Msida, Malta; ^5^Department of Endocrinology, Diabetes and Metabolic Diseases, University Medical Centre Ljubljana, Ljubljana, Slovenia; ^6^Faculty of Medicine, University of Ljubljana, Ljubljana, Slovenia; ^7^Department of Research and Health Statistics, The Centre for Disease Prevention and Control, Riga, Latvia; ^8^European Commission, Joint Research Centre (JRC), Ispra, Italy; ^9^Hub for International Health Research, Perugia, Italy; ^10^Department of Public and Occupational Health, Amsterdam UMC, Amsterdam Public Health Research Institute, University of Amsterdam, Amsterdam, Netherlands

**Keywords:** NCD indicators, EU health information systems, diabetes, quality of care and outcomes, European health data space

## Abstract

**Background:**

The Collaborative Health Information European Framework (CHIEF) is an initiative led by the Joint Research Centre (JRC) of the European Commission to enable a sustainable data collection and production of indicators to monitor and evaluate best practices for people with NCDs.

**Objectives:**

We aimed to support international assessment and comparability of NCD indicators through a taxonomy of NCD indicators, a core set of measurable diabetes indicators and high-level policy recommendations.

**Materials and methods:**

The study is an expert review run by the multidisciplinary expert group “CHIEF-diabetes.dwg” between 2022 and 2024. The group convened on several remote and in-presence meetings. A common set of key references were identified to underpin collective discussions and agree on the fundamental criteria for the selection of indicators. An iterative process was followed to reach agreement and release final recommendations.

**Results:**

A taxonomy of NCD indicators and relevant stakeholders was identified to guide the selection process. A core set of diabetes indicators was agreed, including: diabetes incidence and prevalence rate, retinopathy prevalence rate, major lower extremity amputation incidence rate, blindness rate and rate of end stage renal disease. Measurement issues across chronic diseases and data collection were included in recommendations to the EU.

**Discussion:**

The taxonomies and core set of diabetes indicators identified by the expert group may be used as a proof of concept of a collaborative European framework. The expert group recommended: (a) to determine the purpose of NCD indicators in advance; (b) to prioritise actionable indicators for the high-level governance of chronic diseases; (c) to align with existing standardisation processes; (d) to build on the experience of existing registries; and (e) to align with current efforts to strengthen the EU data infrastructure.

**Conclusion:**

The expert group delivered general guidance on criteria and principles for the selection of NCD indicators, using diabetes as a case study. The core set of six diabetes indicators can be used as a field-test for future reporting initiatives. In this context, disease registries can provide the high-quality data needed to compute reliable indicators. Targeted projects are needed to design a cohesive health information system of NCD indicators.

## 1 Introduction

Agreeing and harmonising health data indicators is challenging in the heterogeneous backdrop of European health service infrastructures. However, standardised indicators are necessary to support measures that would curb the rising number of people suffering from chronic diseases, through the continuous improvement of clinical practice.

### 1.1 NCD indicators and the CHIEF initiative

Measuring clinical outcomes requires feeding data into statistical routines at the local level. This would thereby improve the ability and efficiency of data providers to produce comparable high-quality data, using a coherent infrastructure that can effectively exploit multi-source secondary data uses (1, 2), and serve as a basis for multiple quality improvement strategies (3).

To progressively cover all non-communicable diseases (NCDs), a range of suitable indicators needs to be defined in all their essential components, making the process applicable to other disease domains. The methodology to decide and agree on indicators should be supported by a set of criteria that can be incorporated in the contextual description of an indicator. This approach could adequately address any bias or limitation, dependent upon the data collection modalities and availability of related information.

A systematic approach to indicator selection employs a combination of expert insight and literature review, to which it is possible to add the perspectives of a range of stakeholders, including patients, clinicians, managers, and policy makers (4).

Collaborative efforts result in more relevant questions being addressed and answered. Different types of stakeholders ensure that the selected indicators are meaningful, comparable and actionable across diverse healthcare settings.

The Collaborative Health Information European Framework (CHIEF) launched by the European Commission (EC) services under the technical coordination of the EC’s Joint Research Centre (JRC), is a targeted initiative aimed at strengthening the production of indicators on NCDs in Europe (5).

The aim of CHIEF is to lay out a holistic infrastructure that would enable a sustainable data collection to monitor and evaluate best practices for people with NCDs.

### 1.2 Defining the purpose of indicators

The selection of indicators needs to be preceded by a precise specification of their purpose and scope. In the area of NCDs, the purpose of indicators differs between the macro, meso and micro levels (6, 7).

At the micro level, an indicator encompasses the decision-making process in the provision of care. The focus is on service improvement, small units of analysis and frequent reporting cycles. They are used to inform patients and improve the performance of individual practices and professional teams.

The use of indicators at the meso level enables organisations to recognise positive or negative changes in health care quality. They are used for quality improvement across networks and areas of specialisation.

At the macro level, indicators are focused on outcomes, as they aim to estimate the burden of diseases and quality of life of populations. They are used to steer, manage and evaluate governance and health system interventions.

Defining a precise core set of NCD indicators represents one of the main challenges in the higher goal of building a common EU information system. The difficulties involve agreeing the purpose for which indicators are produced, ensuring their comparability for international benchmarking, and improving their standardisation (deploying guidelines, e.g., ICHOM and sharing knowledge across different professional communities and data systems).

A range of high-level indicators seem to be already accepted for straightforward implementation in NCDs. In prevention, many risk factors are well defined for several diseases. For early detection, screening or staging at diagnosis are also relevant. At population level, prevalence data are widely accepted for short term planning and management purposes, while the prevention of complications and quality of life improvement are considered more actionable than overall mortality.

The increased use of real-world data (RWD) extracted from multiple health-related data sources offers new opportunities to estimate NCD indicators securely at regional, national and international level, using privacy-enhanced statistical methods in context, e.g., federated networks (8).

However, major challenges are still posed by the inconsistent application of FAIR data principles (make data “Findable, Accessible, Interoperable, and Reusable”) and lack of data quality monitoring in the heterogeneous information infrastructure of European countries, hampering the production of unbiased indicators and the correct epidemiological interpretation (9).

### 1.3 Diabetes as a case study

In diabetes, researchers aim to use indicators to improve the understanding of aspects such as disease prevalence, stage of disease, comorbidity, quality of life and the related healthcare requirements, with a view to ascertaining good practices. Indicators can help highlighting high-risk strata that can be effectively targeted by appropriate prevention strategies.

Micro-level indicators focus on individual patient factors, such as blood glucose levels, glycated haemoglobin and lifestyle choices (e.g., diet, exercise and medication adherence). Meso-level indicators examine the role of healthcare organisations, teams, and community resources. Macro-level indicators consider broad societal factors that can impact on diabetes prevalence and healthcare governance, e.g., health policies, socioeconomic conditions, and environmental protection (10).

Using health indicators in diabetes has traditionally worked particularly well, although there is still a lack of consensus on best practices to ensure national coverage, particularly through targeted data collection strategies (e.g., implementing population-based diabetes registries).

To overcome this problem, various projects addressed the implementation of a core set of diabetes indicators, including the health care quality indicators project (11) led by the Organisation for Economic Co-operation and Development (OECD), the global standard set (4) delivered by the International Consortium for Healthcare Outcomes Measurement (ICHOM), the information gateway of World Health Organisation European Region, WHO-EURO (12), the United Nations (UN) Sustainable Development Goals (SDGs) Indicator Framework (13) and the Atlas of the International Diabetes Federation, IDF (14).

In Europe, the EC Directorate for Health and Consumer Affairs (DG-SANCO) funded specific initiatives in the field of diabetes information: EUDIP (15), EUCID (16), ECHI (17), BIRO (18, 19), EUBIROD (20, 21). These projects addressed the definition of a core set of indicators for deriving them.

Despite considerable headway in a notoriously challenging data landscape, there was neither a consistent follow-up nor a sustainable solution proposed for diabetes and other disease domains after this stream of dedicated projects.

### 1.4 Study objectives

The present study builds on previous activities in diabetes, to prove the concept of how a core set of indicators could work across different NCD domains. The approach addresses the need of adding contextual information, provenance, and a quality trail through the processes involved in deriving each indicator.

Our general aim was to provide key recommendations at the EU level for international assessment and comparability of NCD indicators.

The specific objectives of the study were the following:

- to identify a taxonomy that can support choices in the collection and harmonisation of indicators at the EU level;
- to define a basic set of diabetes indicators, including purpose, numerator, denominator, disease classification code and standardisation procedures;
- to produce high-level policy recommendations to improve the international comparability of indicators.

The results shall provide the EU with a platform of NCD indicators for monitoring, benchmarking, auditing, and feedback on quality of care and outcomes.

## 2 Materials and methods

We carried out an expert revision coordinated by the EU-JRC between 2022 and 2024.

The task involved the formation of the CHIEF “diabetes design working group” (CHIEF-diabetes.dwg), an expert group formed by the European Commission to discuss, agree, define and test a basic set of harmonised indicators at the EU level. The other points of focus addressed by the group included: metadata, federated analytics, privacy and ethics and stakeholder engagement. Participating experts included all authors of the present paper.

The qualitative study was conducted through a series of remote evaluation rounds. In particular, two annual in-presence meetings were organised in November 2022 and October 2024 at JRC premises, Ispra, Italy, and an interim meeting was held in the occasion of the World Diabetes Congress in Lisbon, December 2022.

During the 2 years of activity, the regular exchange and update of materials was discussed at regular online meetings, where experts discussed the methodology and state of the art of the study, until the final selection of indicators was made.

### 2.1 Criteria for the selection of indicators

As a first step, experts agreed a common set of criteria to evaluate and prioritise healthcare indicators for international comparisons, drawing on best practices from leading healthcare organisations, including the OECD, ICHOM, WHO, UN SDGs and the EU.

Different criteria were listed in terms of definitions and relevant questions for each candidate indicator. For instance, to align with healthcare objectives, an indicator should be consistent with the stated goals and purposes of comparisons, specifying whether it measures a healthcare outcome, process, or system characteristic.

The criteria used for the selection of diabetes indicators are listed in Table 1.

**Table 1:** CHIEF criteria for the selection of NCD indicators.

**Criterion**	**Definition**	**Questions to ask**
Alignment with healthcare objectives	The indicator should align with the goals of the healthcare comparison	What is the purpose of the comparison (e.g., benchmarking healthcare systems, tracking progress toward goals)? Does the indicator directly measure a healthcare outcome, process, or system characteristic?
Conceptual clarity and consistency	The indicator should be clearly defined and consistently understood across countries	Is there a standardised definition of the indicator (e.g., WHO or OECD definitions)? Does the indicator measure the same healthcare concept in all contexts?
Methodological consistency	The data collection methods should be consistent across countries	Are the same data sources (e.g., national health surveys, hospital records) used? Are the sampling methods, timeframes, and measurement tools comparable?
Data availability	The data should be available for all countries being compared	Is the indicator reported by most or all countries in the comparison group? Is the data available for the same time periods?
Data quality	The data should be accurate, reliable, and complete	Is the data collected using rigorous methods (e.g., validated surveys, administrative records)? Are there mechanisms in place to verify data quality (e.g., third-party validation)?
Contextual relevance	The indicator should be meaningful and relevant in all healthcare contexts	Does the indicator reflect healthcare priorities in all countries being compared? Are there contextual factors (e.g., cultural norms, healthcare system structures) that might affect the interpretation of the indicator?
Comparability over time	The indicator should be comparable across different time periods	Has the definition or methodology of the indicator remained consistent over time? Are historical data available for trend analysis?
Statistical robustness	The indicator should be statistically valid and reliable for comparison	Are there statistical tests to confirm comparability (e.g., measurement invariance)? Is the indicator sensitive enough to detect meaningful differences in healthcare outcomes?
Actionability	The indicator should inform healthcare decision-making or policy changes	Does the indicator provide insights that can lead to actionable recommendations (e.g., improving access, reducing costs)? Is the indicator linked to specific healthcare interventions or policies?
Alignment with international healthcare standards	The indicator should align with global healthcare standards or frameworks	Is the indicator part of internationally recognised frameworks (e.g., SDGs, WHO, OECD)? Does the indicator follow global best practices for healthcare data collection and reporting?

### 2.2 Steps for the selection of indicators

The agreement on the different threads of work and final synchronisation were reached via coordination meetings. In particular, the key criteria and related taxonomies were specified in the first annual meeting organised in 2022 at JRC premises.

On that occasion, the experts agreed to select indicators using the stated criteria in a series of predefined steps:

1. Taxonomies of indicators and relevant stakeholders: to provide a common baseline for the assessment of candidate indicators.
2. Definition of scope and purpose: to clarify the objectives of healthcare comparisons, e.g., benchmarking healthcare systems, tracking progress toward goals, and propose an initial set of countries or regions to be compared;
3. Identification of potential indicators: to compile a list of candidate indicators from relevant healthcare frameworks.
4. Application of the selection criteria: to evaluate each indicator against the stated criteria and taxonomies.
5. Prioritisation of indicators: to rank indicators based on how well they meet the criteria, and select those appearing more suitable for immediate testing within CHIEF.
6. Validation on field: test the selected indicators in a subset of countries, to ensure that they work as intended, and subsequently refine the list based on feedback and results.

The selection of indicators was primarily based on the application of the stated criteria, according to the type of indicator, stakeholders, scope and purpose, for each indicator included in a long list extracted from the standards released by the OECD (22), ICHOM (23), WHO-EURO (12), UN SDGs (13), and EUBIROD (20).

The collective discussion included considerations on data availability and fitness-for purpose for a limited subset of outcomes extracted from the above sources, which were included in a draft report prepared by two task coordinators. The report included a long list of candidate indicators agreed during a series of remote and at the interim in person meeting.

The selection of indicators was completed at the second plenary session, during the annual meeting held at JRC premises in 2024. The panel included experts from different disciplines (including clinicians, internal medicine specialists, public health specialists and experts, data-sciences experts and patient representatives).

At the meeting, the group discussed the contents of the draft and derived a short list of indicators, including only those indicators that were considered eligible in terms of scope and purpose of CHIEF. Each of the other criteria were considered to re-order indicators in the short list, until consensus was reached on the first six to be measured in a “proof of concept.”

The alignment with international projects and availability in diabetes registries was thoroughly discussed for the final selection, including considerations related to the metadata standards and data quality frameworks chosen for implementation. The need to contextualise indicators (i.e., to fully reference and dynamically inspect all components of an indicator) was also debated to ensure consistency with the principles of linked open data, e.g., using semantic linkage, data dictionaries and ontologies (24, 25).

Communication with regional and national diabetes patient forums was guaranteed by the direct participation of experts who were already engaged with relevant networks.

## 3 Results

### 3.1 Taxonomy of NCD indicators

The taxonomy of NCD indicators and related domains identified by the expert group are presented in Table 2. Indicators were classified according to areas, indicators and challenges. The related contents are presented in detail in the following sub-sections.

**Table 2:** Taxonomy of indicators.

Area	Indicator(s)	Challenges
Epidemiology	Prevalence	Multiple sources
	Incidence	Type 1 vs. Type 2 Pregnancy outcomes
	Burden of disease	Cause of death
Prevention	Risk factors	Overlap with cardiovascular diseases
		Nutrition/Mobility
		Mental health/depression
Diagnosis	Timely diagnosis	Screening Retinopathy
	Complication related to the date of diagnosis	
Treatment	Medication	Compliance Metabolic control
	Behavioural Measure	
	Hospital admission	
Outcomes	Amputations	Second/tertiary prevention
	Renal failure	
	Retinopathy/Blindness	

#### 3.1.1 Indicators on epidemiology

Indicators relevant for the epidemiology of a disease involve the calculation of measures, e.g., prevalence, incidence and the burden of disease.

For prevalence, registries can provide an essential tool through the many activities that are carried out on a routine basis (26). However, it is necessary to validate the data across multiple primary data sources. Prevalence data included in the registry need to be used with other sources such as household surveys, insurer or hospital administration, etc. In this way, it is possible to triangulate and construct a realistic picture of prevalence in many countries, the figures of which may be underestimated, due to systematic under diagnosis.

Concerning incidence, while this has been the standard reporting metric for type 1 diabetes, the number of published studies reporting the incidence of type 2 diabetes is relatively small (27). This is also due to the fact that diabetes registries and other stable sources are established most often for Type 1 only.

The data collection required for the calculation of the burden of disease in diabetes may be particularly challenging, due to the limited reliability of mortality data. In fact, unlike oncology and cardiovascular diseases, it has been traditionally rare for diabetes to be recorded as the primary cause of death in official statistics (28). As a consequence, countries are dependent on the quality of coding of additional diagnoses, which needs to be thoroughly assessed, to make results meaningfully comparable (similarly to other NCD domains). Once these figures have been validated, countries can then calculate the burden of disease using methods similar to those applied in cancer.

#### 3.1.2 Indicators on prevention

Regarding prevention, most risk factors are identical to cardiovascular diseases. A positive aspect is that registries can build on what is already known about aspects such as nutrition status and habits, mobility, etc. This information is generally available from elsewhere.

#### 3.1.3 Indicators on diagnosis

The time of diagnosis and how this is meaningfully captured can be influential on the calculation of indicators. As a basic assumption, there seems to be substantial under-diagnosis, with diabetes status usually correctly attributed at a much later stage (29). The group believed that there needs to be ways of creating, as for oncology, some type of staging information that can provide an idea of a potential diagnosis at a much earlier stage.

Part of the available knowledge is screening-based, but not systematic. There are also ethical aspects that must be considered, including the heterogeneity of approaches, making screening in one country difficult to compare to screening in other countries.

A possibility would be for registries to agree on a standard method for estimating the timeliness of diagnosis (30). For instance, in most cases, registries include information on negative outcomes, e.g., retinopathy, amputation and renal failure: the time taken in general to develop a given complication could be used to validate the timeliness. For example, the number of new diagnoses made per year in Latvia, which is approximately equal to 6,000, may translate into a percentage of negative outcomes foreseen within a certain period. The latter could then be used as a proxy for how timely the diagnosis has been.

#### 3.1.4 Indicators on treatment

Indicators on treatment usually have a great deal of information available. At a system level in diabetes, one major aspect of interest is metabolic control, which could be basically calculated if all registries measure the key variables such as HbA1c level in a comparable way. This measure should be consistently monitored over a certain period in a specific population, with follow-up requiring precise explanations on why metabolic control has not been achieved (e.g., lack of compliance, behavioural problems etc., all aspects which may not be directly related to the specific medication).

These insights were discussed over 10 years ago at the OECD, suggesting the use of hypertension medication given to patients with diabetes as the only element that could be meaningfully compared across more than 10 countries (31).

A useful variable that has been analysed in depth (due to its wide availability) is avoidable hospital admissions. The unplanned admissions for diabetes (if diabetes is properly recorded in the discharge databases) can be considered avoidable and can be used to differentiate between planned and unplanned cases.

The OECD uses measures of avoidable admissions for other chronic diseases, e.g., COPD, asthma, and chronic heart failure (11). Countries found it to be quite meaningful, since it highlights the product of primary care more than the hospital. Such an indicator may be a potentially good candidate for a range of chronic conditions.

Further information about secondary and tertiary prevention is also available from diabetes registries.

Different NCDs often share common underlying pathophysiological mechanisms and are therefore sometimes treated with the same medications. Currently, such multipurpose medications for diabetes (e.g., GLP-1 agonists, SGLT-2 inhibitors) are primarily analysed within a single disease domain or associated disease registry. CHIEF will offer a platform to widen the perspective on treatment dynamics, by combining common data elements from various disease domains. It will also facilitate knowledge exchange, cross-disciplinary interpretations and data reuse among stakeholder communities from different medical specialities. Such an approach will also enable the development of indicators for assessing diverse elements associated with various NCDs simultaneously, while expanding upon traditional methodologies.

#### 3.1.5 Indicators on outcomes

Three outcomes have been used in diabetes to assess how well countries are performing (11):

- Amputation rates, for which there is already international comparative work and criteria that are regularly applied (32–35).
- Renal failure, which requires specific attention, provided that a certain level of standardisation is present in diabetes registries.
- Retinopathy and blindness, with the same caveats.

New perspectives in the calculation of the above indicators are worth exploring, including:

- Access to healthcare services, which is relevant to ensure that certain aspects (e.g., access to diagnosis or access to treatments) are not associated with geographical or social characteristics. In fact, it has been found that large differences exist between rural and urban populations (36) or in the financing of certain therapies (37). Thus, it would be possible to stratify indicators using access to services to highlight such effects.
- Health inequalities (similarly to oncology), where the existing indicators look at disparities in specific strata, e.g., variability in geographical distribution and/or socio-economic status. An early assessment is required to check the extent of coverage and ease of obtaining such information from diabetes registries. Several registries in Europe have the means of performing the necessary data linkage (38).
- Patient-reported outcome measures (PROMs—outcome indicators that matter most to the patients) and Patient Reported Experience Measures (PREMs—measures that report on the individual experiences of the person in their encounters with the health care system) are in phase of implementation (4). Regional and national information systems are encouraged to include such measures in information captured on patients. A discussion is ongoing on how this could be integrated in regular data collection (39). There are only few examples of standardised datasets adopting PROMs for their system of indicators in diabetes (40).

#### 3.1.6 Taxonomy of NCD stakeholders

The use of data by patients, health professionals and policy makers is critical to enhance the successful use of indicators. However, access to different silos of information is heavily dependent on the capacity to link across data sources.

To help clarify the needs of different classes of users, CHIEF-diabetes delivered an initial taxonomy of stakeholders related to the data collection process (see Table 3).

**Table 3:** Taxonomy of stakeholders.

Stakeholders	Context	Databases
Citizens	Access	Devices
Patients	Shared decisions	EHR
Health care Professionals		Registries
Management	Labour shortage	AI
Financing	Value-based health care	PREMs, PROMs
National Policy	Health Systems Performance Assessment, Inequality	Aggregate/Linked/Federated Data
European Union		

Three different dimensions were considered relevant for the use of indicators: the type of stakeholder, the related context and the relevant data source.

The use of indicators by citizens was considered relevant for access, which can be vastly improved by making use of personal devices (e.g., smartphones or wearable systems). Patients and healthcare professionals can use indicators to share decisions, capturing information via electronic health records (EHRs) and disease registries.

On the other hand, managers can be interested in using health information to optimise the availability of the skilled workforce, using modern tools, e.g., artificial intelligence (AI). At a European level, there is a pressing need to optimise the digitalisation of health data as a means to improve efficiency in health systems. In many countries of Europe, this triggered legislative reform and service redesign, particularly after the COVID-19 pandemic, in response to one of the largest EU investments including the aims of enhancing public trust and data access in support of digitalisation (41).

At the higher governance level, finance officers can use PREMs and PROMs to implement value-based health care, while international organisations can use indicators for health systems performance assessment (HSPA) and the control of inequalities, using aggregated, linked or federated data.

At all levels, it could be possible to increase the interaction between patients and their healthcare providers, to strengthen the contents of disease registries. However, these data sources must be linked with other sources, e.g., mortality, administrative and health insurance data (where necessary). There is a further need to link registries with household surveys. In this complex scenario, integrating registries with digital sources and devices (e.g., electronic health records and smartphones), provided that this is done in a secure environment, may provide direct benefits to all people with NCDs.

Currently, there are limited possibilities to perform data linkage across the board, but a coherent information infrastructure needs clinical data and the level of competence that is frequently present only among experts involved in disease registries (42). In this regard, the experience of registries may be highly pertinent for the ongoing plans of building an ecosystem with higher linkage capability within the European Health Data Space (EHDS).

#### 3.1.7 Indicators in diabetes

On the basis of the above criteria and associated taxonomies, CHIEF-diabetes finally selected the following six diabetes indicators:

- Incidence
- Prevalence rate (%)
- Complications at diagnosis—retinopathy prevalence rate (%)
- Major lower extremity amputation incidence rate (per 1,000 persons with diabetes annually, aged ≥15 years)
- Blindness rate (per 1,000 persons with diabetes)
- Chronic renal failure rate—end stage renal disease (ESRD) (per 1,000 persons with diabetes)

The definitions of all measures in terms of indicator, purpose, numerator, denominator and coverage are presented in detail in Table 4.

**Table 4:** Diabetes indicators.

Indicator	Purpose	Numerator	Denominator	Coverage
Incidence	Monitoring of incidence (crude and age standardised) of diabetes mellitus will provide insight into the burden of disease (size of the problem) and trends during the time as well as enabling public health workers to monitor the diabetes (as an example of any NCD) epidemic and policy makers to plan health resources. Age distribution needs to be considered, so crude rates and age standardised rates will be calculated. Crude rates will serve for the planning of current health resources while age standardised rates will allow comparison between time and countries. This group of indicators is defined for all age groups and in total consist of 6 parameters.	Number of newly diagnosed persons with diabetes (all ages) in year X. Number of newly diagnosed persons with type 1 diabetes (all ages) in year X. Number of newly diagnosed persons with type 2 diabetes (all ages) in year X.	Total number of people in country in year X – total number of persons with diabetes in country in year X-1. (mid-year estimate of the resident population)	Population aged 0 and older. All primary healthcare units/centres, acute care hospitals, including public and private hospitals that provide inpatient care and registry data.
Prevalence rate (%)	Monitoring of prevalence (crude and age standardised) of diabetes mellitus will provide insight into the burden of disease (size of the problem) and trends during the time. Age distribution needs to be considered so crude and age standardised rates will be calculated. Crude rates will serve for the planning of current health resources while age standardised rates will allow comparison between time and countries. If possible, the whole population should be included. This group of indicators is defined for all age groups and in total consist of 6 parameters.	Number of persons with diabetes (all ages) in year X (end of year); Number of persons with type 1 diabetes (all ages) in year X; Number of persons with type 2 diabetes (all ages) in year X.	Total number of people in country (all ages) in year X (mid-year estimate of the resident population).	Population aged 0 and older. All primary healthcare units/centres, acute care hospitals, including public and private hospitals that provide inpatient care and registry data.
Complications at diagnosis: retinopathy prevalence rate (%)	Retinopathy prevalence in persons with newly diagnosed diabetes mellitus will provide insight into diabetes screening in country revealing its efficacy as well as duration of unrecognised disease in population.	Number of persons with diabetic retinopathy at diagnosis (all ages).	Total number of incident persons with diabetes in country in year X as end-year estimate of the resident population with incident diabetes.	Population aged 0 and older. All primary healthcare units/centres, acute care hospitals, including public and private hospitals that provide inpatient care and registry data.
Major lower extremity amputation incidence rate (per 1,000 persons with diabetes annually, aged ≥15 years)	Lower extremity amputation incidence rate monitoring will provide insight into the potential of health and quality-of-life improvement and the potential for the reduction of healthcare costs. Regular screening to detect people at risk, screening of those at risk as well as those with foot ulceration may reduce number of amputations as well as cost of care since foot care is proven to be a cost-effective intervention in diabetes care. Incidence rate will be calculated using population of persons with diabetes to evaluate amputation risk in the reference population and furthermore to avoid bias caused by increased diabetes prevalence due to lower rates of undiagnosed cases (e.g., resulting from better screening). A major lower limb amputation as defined by the Lower Extremity Amputation Study Group is the loss in the transverse anatomical plane at or proximal to the ankle joint.	Number of persons with diabetes aged ≥15 years with major lower extremity amputation in year X.	Total number of persons with diabetes aged ≥15 years in country in year X.	Population aged 15 and older. All acute care hospitals, including public and private hospitals that provide inpatient care and registry data.
Blindness rate (per 1,000 persons with diabetes)	Blindness rate monitoring will provide insight into the burden of microvascular complications in the population of persons with diabetes of the country. Monitoring will further provide insight into the potential of health and quality of life improvement and the potential for a reduction of healthcare costs (since regular retinopathy screening is proven to be a cost-effective intervention in diabetes care). Incidence rate will be calculated using the population of persons with diabetes in order to evaluate risk in the reference population and furthermore to avoid bias caused by increased diabetes prevalence due to lower rate of undiagnosed cases (e.g., resulting from better screening). Numerator – number of blind persons with diabetes (all ages) in year X.	Number of patients with diabetes and blindness or low vision.	Total number of persons with diabetes in country in year X.	Population aged 0 and older. All primary healthcare units/centres, acute care hospitals, including public and private hospitals that provide inpatient care and registry data.
Chronic renal failure rate – end stage renal disease (ESRD) (per 1,000 persons with diabetes)	ESRF rate monitoring will provide insight into the burden of microvascular complications in the population of persons with diabetes of the country. Monitoring will further provide insight into the potential of health and quality of life improvement and potential for the reduction of healthcare costs and also facilitate planning of health resources (dialysis and transplantation). End-stage renal disease (ESRF) is defined as a previous renal transplant or eGFR < 15 mL/min per 1.73 m2 or on dialysis. Incidence rate will be calculated using the population of persons with diabetes to evaluate risk interference population and furthermore to avoid bias caused by increased diabetes prevalence due to lower rate of undiagnosed cases (e.g., resulting from better screening).	Number of persons with ESRD and diabetes (all ages) in year X.	Total number of persons with diabetes in country in year X (end-year estimate of the resident population with diabetes, i.e., persons with listed diagnosis code for diabetes [ICD-10-WHO E100-E119, E130-E149] or persons from a disease registry).	Population aged 0 and older. All primary healthcare units/centres, acute care hospitals, including public and private hospitals that provide inpatient care and registry data.

Age- and sex-adjusted rates need to be calculated for all indicators and the EU population needs to be used during standardisation. Further details on the coding and calculation of each indicator are included in Supplementary Data.

## 4 Discussion

### 4.1 Selecting indicators: purpose, level and standardisation

The present paper has explored some fundamental concepts required for the design and development of a coherent system of NCD indicators in the EU.

The multidisciplinary expert group assembled in CHIEF considered the purpose of each indicator as a critical prerequisite for any practical implementation. In fact, if the purpose is not clear, it would be impossible to develop a meaningful indicator. Designing an information system, without mentioning at which point of the management cycle an indicator will be used—e.g. improving performance benchmarking or evaluating a specific pathway of care—makes it questionable to judge how useful it could be.

The panel agreed that it is essential to clarify at which level of the health system an indicator might be used. The selection of indicators can be made at different levels: (a) micro-level, to optimise health care provided to the patients; (b) meso-level, to evaluate how an organisation performs according to specific targets; (c) macro-level, addressing international comparisons for overall health improvement. In the context of CHIEF, the expert group considered macro-level indicators more appropriate, as they can facilitate comparability and benchmarking within and across EU Member States.

Judging upon which indicator is more appropriate for the macro level was based on the examination of the most current literature and a set of use cases proposed by the experts. The final choice was aligned with publications by the IDF and OECD, for which full specifications of ICD codes have been also given. Greater divergence may arise from different classifications, where precise specifications may not necessarily exist in the reference literature.

The panel considered the heterogeneity of data sources as a critical aspect influencing the selection of indicators. In this regard, the experience of cancer care may help in understanding how to improve the standardisation process, as well as what to avoid hampering international comparisons. That is particularly relevant in the coordination of CHIEF by the JRC, as this institution maintains also the European Cancer Information System, in collaboration with an international network of registries.

Based on these considerations, experts discussed the positive role of existing networks and international organisations, e.g., ICHOM, for the production of technical guidelines that can improve data standardisation. In this context, diabetes represents an ideal point of reference, given the presence of well-established data sources and diabetes registries (38).

The panel also noted that the development and implementation of a common agenda for HSPA in the EU conveniently promotes the use of large databases for the sake of producing macro-level indicators. However, several countries have not yet implemented these concepts and need still to develop their data systems.

Disease-oriented initiatives, e.g., the EU “beating cancer” plan, triggered the production of country profiles including the concept of inequality registries, where targeted outcome measures are checked for heterogeneity between different subgroups, by socio-economic status (43). More recently, joint actions such as JACARDI (44), due to its collaborative scheme across diabetes and cardiovascular diseases, can help coordinate information exchange between those involved, offering substantial resources that can mobilise actions for strengthening the data infrastructure.

The final selection reflects recent evidence regarding the feasibility of indicators, but also aims to strengthen the data collection of “hard outcomes” e.g., blindness and retinopathy, which may reflect healthcare access rather than true burden. Specific mitigation strategies shall be considered in these cases.

In the case of blindness, although no dataset may be considered 100% complete, it is usually considered a condition that should be duly reported. There might incidentally be a different propensity and access to specific interventions for certain categories of patients. As a mitigation strategy, the analyst would need to estimate the fraction of those hard to reach, to assign a set of higher weights to those presenting similar patterns among those included in the calculation of the indicator. These types of problems are frequently managed by statistical methods, e.g., propensity scores, capture/recapture or weighted regression.

With regards to retinopathy, the panel proposed to measure its prevalence at diagnosis—when by definition the patient is already in charge of a health care provider—as a proxy of the time lag between diabetes onset and diagnosis (45).

### 4.2 Diabetes as a case study for NCDs

In the field of diabetes, a great deal of work has been already performed for the selection of indicators. However, many critical aspects still need to be considered in prioritising their implementation ensuring that they can be equally applied in different contexts.

The panel agreed a point of interest frequently understated is the effect of bias generated by inaccurate time of diagnosis on outcome indicators (46). People with longer duration of diabetes have a higher likelihood of complications, which can be difficult to capture using data sources that are particularly prone to under diagnosis, generating misleading interpretations in terms of risk assessment.

For this reason, experts highlighted the importance of having a point of early detection, where the severity of the disease can be carefully examined, allowing to control the accuracy of the time at diagnosis at an early stage.

The choice of different measures of incidence and prevalence aligns with this advice, as they may also have relevant impacts, given the different targets and meanings in terms of decisions for public health action—the impact of eye disease in diabetes being a case in point (47). Incidence may represent an important aspect to consider in relation to the specification of screening strategies. On the other hand, prevalence data may be more directly useful for short-term planning and management purposes, as they can provide input for the rapid organisation of health services (e.g., five-year survival rates for cancer).

From an epidemiological perspective, capturing greater prevalence may represent a positive aspect, since it translates to more cases to treat, and lives that can be saved. In cancer, greater prevalence can also mean having been effective with treatment (i.e., people are living longer). However, quality of life ought also to be monitored alongside. COnversely, improving screening in chronic diseases means increasing the incidence through improved diagnosis. In these cases, preventing complications and long-term survival by monitoring quality of life may be more actionable than overall mortality.

The expert group noted that diabetes-related indicators are convenient to use at different levels, as they can be measured on a county, regional or national level. Most databases are already in place, although not always directly usable, as they need to be properly identified, allowed to be accessed, and quality-controlled.

Better understanding of data quality is also important from a health care perspective. Accurate data can drive screening campaigns, capturing more people with less advanced disease. These types of mechanisms can be improved by targeting specific indicators that can flag high risk patients.

For this reason, the expert group selected the indicator of retinopathy at the time of diagnosis as a good proxy for staging of the disease. By monitoring the eyesight of people with diabetes at the time of diagnosis in the registry, epidemiologists can estimate the accuracy of the time of diagnosis and compare it across different countries.

The panel also recognised the need to adopt different types of indicators for different NCDs. For instance, blindness, chronic renal failure and amputations may equally represent intermediate indicators in diabetes, similarly to five-year survival in cancer. The choices made in CHIEF were based on the highest priority assigned by experts. However, more complete reports may include multiple options, particularly when more indicators can be considered immediately actionable by policy makers.

### 4.3 Perspectives of clinical and epidemiological interest

CHIEF-diabetes discussed other areas that would be most promising to explore for a road test of NCD indicators in multiple data sources.

There was a general recognition that the interest of all relevant stakeholders would be significantly raised by covering also the meso and micro-level. This would allow cohesive engagement of policy makers, researchers, data custodians, health professionals and people with chronic diseases. However, the expert group agreed that at an early stage it would have been easier to focus on the macro level only.

Among other areas of interest, the expert group highlighted pregnancy and mental health, although they both present challenges in terms of feasibility and stage of maturity of structured data collections.

Incidence in pregnancy can be monitored over a timeframe where blood control is part of routine care and it is thereby possible to identify relevant changes in glucose metabolism. Women may be at higher risk of developing diabetes, and there is increasing evidence on how this can be detrimental also for newborn, not only in relation to metabolic disease, but also to other chronic diseases (48). Unfortunately, the current surveillance systems include limited specific information for international comparisons.

In mental health, various attempts have been made using other data sources, but the difficulty lies in harmonising the available information for meaningful international benchmarking. For instance, there has been relevant work on ultimate outcomes (e.g., suicides), which would not be easy to replicate (49). Administrative data of people admitted to mental-health institutes are available (e.g., OECD indicators for people with bipolar disorder and schizophrenia), which would enable linking between those diagnosed with a certain disease and their mortality data (50). Other indicators may signal the role of mental health on life expectancy, as the excess mortality in those groups can be very high. These issues have triggered activities in the UK to improve concerted efforts to gain better information (51).

Overall, compiling relevant and comparable indicators for mental health has been notoriously difficult and, despite the many attempts over the last 20 years for obtaining good benchmarking information, there is still not a clear framework. Part of the reason is that mental health is not one disease but covers a whole spectrum of diseases (ranging from schizophrenia through bipolar disorders and many other aspects such as depression, anxiety disorders and substance abuse). Despite the fact that many public health institutes are collecting data using surveys and volume data from consultations, admissions, and the time lag between duration of admission and virtual consultations, there is still considerable work and agreement needed.

Neurodegenerative diseases could be another highly relevant area to be explored in the broader field of mental health, given the evidence of the relation between degenerative inflammatory processes (more recently related to COVID-19), brain fog and the cognitive symptomatic decline (52). Additional evidence emerged regarding changes to the brain structure and function, which can be related to diabetes (diabetes is a known risk factor for dementia, especially if it manifests in middle adulthood) (53). Although promising, this area may be not be sufficiently developed to be tackled immediately.

Similarly, there has been substantial interest on exploring the link between depressive symptoms and diabetes via PROMs, thereby helping identify characteristics that predispose people with diabetes to depressive symptoms, which may have significant impact on health outcomes. The ICHOM standard set caters for this information, but the underlying data infrastructure would have to be sustainable (4). This might be possible at a practical level by exploiting medical devices and mobile applications, which can incorporate relevant data captures.

The panel agreed that it would provide greater benefit and flexibility to wait until PROMs could be integrated into the personal health record system, which is currently under test within research projects. Diabetes registries could then link such information with other data sources to deliver more informative indicators, while improving care at the individual level (54).

### 4.4 Towards a cohesive EU framework for NCDs indicators

The most significant challenge for NCDs indicators pertains to the accessibility and harmonisation of primary data, which has been a central interest of CHIEF-diabetes.

Despite its critical importance in enhancing insights into healthcare delivery and outcomes, the harmonisation of primary data use at the EU level has often been overshadowed by secondary applications, such as research and policy development. Nevertheless, this situation has the potential to evolve rapidly with the ongoing EHDS initiative.

The EHDS (55), particularly through the provisions outlined in Article 14, aims to facilitate access to priority categories of personal electronic health data for primary use, which has historically been overlooked by analysts, registry holders and EU-funded projects. The implementation of the EHDS presents a significant opportunity to create innovative, highly accessible indicators that could be uniformly applied across the EU. Active collaboration between different health systems may help building a robust and interoperable health data ecosystem through seamless data conversion and integration across both statistical and clinical domains.

Further harmonisation may enhance the usability of health indicators, improving data consistency, accessibility, and overall utility. Additionally, it will significantly reduce the burden on healthcare providers, as they will only need to enter data once to make them accessible across a unified framework.

The CHIEF experts focused their debate on a system level, where indicators are mainly used for governance and quality improvement. The group deliberately steered away from structural indicators (e.g., the number of diabetes nurses etc.), as they felt this would distract from the goal of having process and intermediate outcome indicators immediately available. Similarly, the expert group did not involve cost-related indicators targeted by incentive programs (e.g., pay for performance), which may not be equally present in other countries. In fact, several countries include diabetes in bundle payment structures, to incentivise the move from hospital-based diabetes to the local services. However, these aspects are still too specific to represent a common EU priority.

For the implementation of chosen indicators, the expert group considered using disease registries as a strategic element for the construction of a cohesive EU information system for NCDs. In particular, experts highlighted the potential improvements that could be gained by cross-linking different registries across diabetes, cardiovascular and respiratory diseases (e.g., chronic heart failure, chronic obstructive pulmonary disease and asthma). Registries generally operate as communities of practices, where the clinical knowledge is widely present and participants are well aware of the evidence-base and use of relevant NCD indicators. The expert group recognised the need of bridging such valuable knowledge across domains.

To prove the concept of CHIEF in a pilot study, it was considered particularly useful to use different registries to validate the time of diagnosis, as data could be linked to compute the time lag until the occurrence of complications. In this perspective, the case of chronic obstructive pulmonary disease (COPD) seems particularly interesting, considering the range of problems that can be potentially attributed to long COVID. The data on COPD are reasonably well coded and although the registers might be limited, given the attention on COVID and pulmonary diseases, it could be interesting to explore the feasibility of such investigation.

In diabetes, experts agreed that using registry data can be essential to ensure the rapid calculation of indicators used for the different purposes of international comparability, analysis of time trends, health-care resource-planning and forecasting of future health-care needs.

However, relevant limitations may hamper their use such as lack of completeness and representativeness of the data. Ideally, a registry should include all patients with diabetes in the country or region. However, this is rarely the case (38). To ensure representativeness, registries should include data from different healthcare settings (primary, secondary and tertiary) and from different parts of countries or regions. Further limitations include the potential bias induced by specific strata with different accessibility to healthcare and a different likelihood of being included in the registry, as well as the possible lack of standardisation of clinical and laboratory parameters in routine clinical practice. This is not usually an issue when assessing specific outcomes (e.g., amputations, end-stage renal disease or mortality), but may be the case, for example, when assessing blood pressure, HbA1c or lipid control. In some countries, indicators, e.g., incidence or prevalence are calculated using a proxy, based on the level of adherence to specific care models and/or insulin use, rather than a direct calculation from registry or RWD.

### 4.5 Stakeholders participation

The methodology used to reach consensus among experts for the selection of indicators was limited to participants in the expert group, including representatives of patients’ associations.

Future efforts should establish formalised collaboration mechanisms to facilitate the timely integration of diverse stakeholders by enabling open commentary during the consensus-building process itself.

Such an approach closes the feedback loop by directly involving frontline providers and patients, thereby ensuring that the real-world efficacy of the results is evaluated by those on whom they impact most. This is essential for incorporating expressed needs and for continually improving the proposed measurement methods.

The inclusion of stakeholders in the management of diabetes clinical registries seems limited and highly diverse (38), while the active use of diabetes indicators appears practically unexplored (with the exception of personal clinical measurements). This situation is likely mirrored in other NCD data governance models. Research into methodologies for the effective and equitable engagement of both patients and healthcare professionals in the production and use of indicators is essential to fully leverage their insights and maximise the value of these data sources.

To build capacity for representative participation in the European Health Data Space (EHDS), healthcare providers are increasingly engaging their professional societies in the ground work needed to ensure their involvement with data sharing (56).

By advocating an improved usability of their everyday tools and the adoption of the ‘once-only principle,’ the same institutions aim to prevent duplication and reduce administrative burdens stemming from secondary use obligations, thereby enhancing the creation of usable data (57).

These bottom-up efforts are complemented by dedicated EU education initiatives in data management, such as the Xpanding Innovative Alliance (XiA) project (58), alongside their involvement in other EHDS initiatives. Consequently, insights generated at the healthcare provider level will create a feedback loop that strengthens their position and enriches the entire lifecycle of indicator development and usage.

## 5 Final recommendations on NCD indicators

All the above aspects were taken on board to provide recommendations for the definition and production of indicators that can overcome the stated limitations in a systematic manner.

The following five high-level policy recommendations, with associated steps for implementation, have been drawn up for the continuous improvement of indicators for diabetes and associated chronic conditions, and more in general for the selection of core indicators in NCDs:

1. *Determine the purpose of NCD indicators in advance*: consider aspects. e.g. performance improvement of healthcare professionals, providing better information to patients, or pursuing continuous quality improvement across networks and different areas of specialisation.
  Steps for implementation: identify communities of practice (specialty networks, scientific associations, patients’ organisations, etc) from which members of competent panels can be drawn to collaborate on common topics; group experts by clusters and tasks to review indicators according to their purpose and scope.
2. *Proritise indicators that are actionable for the high-level governance of chronic disease related healthcare services (input, throughput, output/outcomes) and apply a health services approach in a way that is broader than the classical public health/epidemiological approach*: indicators at the macro level could be used to steer, manage and evaluate the health system interventions and thus are suitable for international comparability of health system.
  Steps for implementation: form study groups including experts skilled in health system policy (regional, national and international); evaluate, score and rank chronic disease indicators selected by communities of practice, taking actionability at macro level as a prioritised criterion; design and support field test of indicators with proper level of funding.
3. Align with existing standardisation in the specific chronic disease area (professionals, science, national, etc.) and indicators already in use by international bodies, providing the necessary contextual information for direct implementation in EHDS-ready information systems: consider the current data collection systems implemented by institutions (e.g., EUROSTAT, OECD, WHO, IDF and global networks such as ICHOM) and identify all common data elements (CDEs) required to implement definitions into a system aligned with the most current technologies and regulations.
  Steps for implementation: start projects to assemble multidisciplinary teams composed of qualitative and quantitative experts, e.g., clinicians, epidemiologists, statisticians, legal and IT experts; identify relevant data sources and coordinate activities to analyse them and derive the CDEs required for calculations; design and develop open software to access and process data sources in a secure infrastructure.
4. *Build on existing European registry initiatives to accelerate a short-term collection of comparable data, making sure that the meso and micro levels are included in the overall framework, to engage all relevant stakeholders*: draw on exemplary cases from pioneering initiatives run by the European Commission, addressing the comparability of indicators for major and chronic diseases among member states. An example in the field of diabetes has been provided by the DG-SANCO co-financed stream of three consecutive projects led by the EUBIROD network, leading to specific data standards (59).
  Steps for implementation: establish coalitions of disease registers, supporting the creation of new ones whenever possible and not existing, and use specialised skills to deliver a broad range of indicators; align with standards and the choice of macro-level indicators made at the outset, using the software specifically developed for the scope; expand on a choice of targeted meso and micro-level indicators, engaging health professionals and the patients in high quality data collection and reporting.
5. *Seek alignment with the broader strengthening of the data infrastructure in EU member states*: consider the requirements set by the new regulations of the EHDS as an occasion to map diverse datasets and research initiatives.
  Steps for implementation: map all information to common data dictionaries validated in multiple settings; exploit the interoperability of disease registries to collect complete data on comorbidities (59); take into account all relevant privacy regulations (60); apply open standards for continuous monitoring and improvement, allowing external evaluation and reuse of any statistical procedure involved (61); embed data quality assessment in the routine reporting of NCD indicators; ensure adequate contextualisation of all indicators using tools, e.g., meta-registry. to ease interpretation and direct comparison against recognised international standards.

These recommendations will be taken on board for the final report of CHIEF, along with other results obtained by other strands of the work of the expert group.

### 5.1 Strengths and limitations of the study

The present study has several strengths that can offer added value to the debate on the evolution of the European health information infrastructure:

- Addresses a topic that is timely and relevant, addressing the need for harmonised NCD indicators, matching the most recent initiatives promoted by the European Commission and other international institutions, e.g., the OECD and WHO Europe.
- Delivers a clear taxonomy of indicators and stakeholders, followed by a structured presentation of the results.
- Selects six core diabetes indicators that are well justified and practical for comparability.
- Provides a set of high-level policy recommendations, each associated to well specified implementation steps, aligned with ongoing EU initiatives (EHDS in particular).

At the same time, the study also presents relevant limitations that are worth to be addressed, including:

- there was a limited number of experts participating in the study, mainly including contributors to the EUBIROD network, which could make the results prone to confirmatory bias.
  However, the group was knowledgeable of the topic and highly multidisciplinary, making the outputs generated by the debate particularly robust. In particular, indicators were selected during a plenary session, in which all decisions were collectively taken by a panel including representatives of people with diabetes, clinicians with direct responsibilities on the provision of health care, and coordinators of international activities involving policy makers.
- there was only one disease domain covered in the field of NCDs. Exploring the implementation of our ideas across all NCDs would require a full blown project that goes beyond the scope of the CHIEF initiative.
  However, this paper has delivered a useful set of taxonomies that can be directly used as a method to derive short lists of indicators for other chronic diseases. The methods outlined are clearly general, and may provide guidance to standardise approaches across NCDs, contributing to a framework that can more directly cross-reference systems of indicators.
- there was no quantitative scoring attached to each diabetes indicator identified, making the ranking and selection process difficult to assess. As a matter of fact, the shortlist and final selection of indicators were produced interactively by the experts during the meetings, without any quantitative evaluation. Therefore, the final list reflects their views and final agreement, following all considerations included in this report. This limitation may induce different types of potential confirmation bias that can be reflected in the choice of indicators.
  However, the experts were from different backgrounds, covering the breadth of professional skills that are necessary to reflect current challenges in the field of chronic disease indicators, in particular data quality and accessibility. These criteria are also the most limiting in terms of feasibility and require careful individual assessment, especially for indicators and data elements that have not been used previously. The lack of precise information in relevant data sources (38) hampers the possibility of stratifying their value based on quantitative measures and forces a high reliance on subjective expert opinion. This problem should be resolved by the establishment of mechanisms for EU quality labels under the EHDS, which will enable a quantitative comparison of the proposed criteria for the selection of indicators.
- the technical guide for the calculation of indicators is only provided as a general reference and may not be detailed enough to perform calculations. In particular, the range of classifications covered only includes WHO-ICD-10 and ACHI codes. Mapping to other systems, e.g., ICD-9-CM, ICD-11, SNOMED and READ, would be immediately needed through other sources. This can be facilitated by automated procedures available to map across different classification systems, which can be embedded in the contextualisation aspects. This issue is particularly critical in terms of geographical variation of the results, as there is known cross-country variability in coding, underdiagnosis and registry completeness, which is also correlated to the use of specific classification systems (34, 35). Moreover, data collection requirements and statistical techniques for the standardisation of indicators have not been addressed.

However, these aspects will be covered by other tasks of CHIEF that will be published successively.

## 6 Conclusion

This paper has provided guidance on a set of criteria and principles for the selection of NCD indicators, using diabetes as a case study.

The core set of six indicators identified by CHIEF-diabetes have been selected as a basis for the next phase, in which the outputs of this study can be used as a field-test for future reporting initiatives. We have provided further details on their purpose and the requirements for their calculation.

A set of indicators providing insight in burden, early detection, treatment and outcome according to the taxonomy outlined here, can enable international comparability through the collaboration of different data sources within and across diseases. In this fragmented context, disease registries may be particularly helpful to provide an immediate startup.

The construction of relevant interconnection tools, e.g., a metadata-registry and an indicator contextualisation framework, can help overcome major challenges in the usability and interpretation of NCD indicators which have previously difficult in European projects.

Although focusing only on diabetes at this stage, the process nevertheless has a general focus, and can represent a relevant starting point to advance the work of CHIEF within the future EHDS.

## Data Availability

The original contributions presented in the study are included in the article/supplementary material, further inquiries can be directed to the corresponding author.
