# Supplementary material for: A taxonomy of indicators for non-communicable diseases: agreement, definition, and contextual description using diabetes as a case study
Source: Front Public Health. 2025 Oct 13;13:1685731. doi: 10.3389/fpubh.2025.1685731 (PMC12554757; doi:10.3389/fpubh.2025.1685731)
Supplement: Supplementary file 1 [file Table_1.docx]

**Supplementary Data**

**Diabetes indicators selected by CHIEF-diabetes.dwg**

## CHIEF-diabetes Indicator 1: Incidence

| **Numerator** | **Denominator** |
| --- | --- |
| **1. Number of newly diagnosed persons with diabetes (all ages) in year X.**  Number of patients (unique person identifier) with primary healthcare unit/centre visit or hospital discharges and any listed diagnosis code for diabetes (ICD-10-WHO E100-E119, E130-E149) or registry patient that were not diagnosed previously.  *Exclusion criteria:* transfers from a different hospital, non-resident patients, cases with MDC 14 or pregnancy, childbirth and puerperium codes in any field (ICD-10-WHO A34, F530-F539, O00-O99, Z320-Z392, Z640). | Total number of people in country in year X – total number of persons with diabetes in country in year X-1. (Mid-year estimate of the resident population – number of persons with diabetes in previous year). |
| **2. Number of newly diagnosed persons with type 1 diabetes (all ages) in year X.**  Number of patients (unique person identifier) with primary healthcare unit/centre visit or hospital discharges and any listed diagnosis code for diabetes type 1 (ICD-10-WHO E100-E109) or registry patient that were not diagnosed previously  *Exclusion criteria:* other types of diabetes E110-E149, transfers from a different hospital, non-resident patients, cases with MDC 14 or pregnancy, childbirth and puerperium codes in any field (ICD-10-WHO A34, F530-F539, O00-O99, Z320-Z392, Z640). |  |
| **3. Number of newly diagnosed persons with type 2 diabetes (all ages) in year X.**  Number of patients (unique person identifier) with primary healthcare unit/centre visit or hospital discharges and any listed diagnosis code for diabetes type 2 (ICD-10-WHO E110-E119) or registry patient that were not diagnosed previously  *Exclusion criteria:* other types of diabetes E100-E109, E120-E149, transfers from a different hospital, non-resident patients, cases with MDC 14 or pregnancy, childbirth and puerperium codes in any field (ICD-10-WHO A34, F530-F539, O00-O99, Z320-Z392, Z640). |  |

## CHIEF-diabetes Indicator 2: Prevalence rate (%)

| **Numerator** | **Denominator** |
| --- | --- |
| **1. Number of persons with diabetes (all ages) in year X (end of year)**  Number of patients (unique person identifier) with primary healthcare unit/centre visit or hospital discharges and any listed diagnosis code for diabetes (ICD-10-WHO E100-E119, E130-E149) or registry patient (excludes – transfers from a different hospital, non-resident patients, cases with MDC 14 or pregnancy, childbirth and puerperium codes in any field (ICD-10-WHO A34, F530-F539, O00-O99, Z320-Z392, Z640). All persons deceased till X-1 year need to be excluded. | Total number of people in country (all ages) in year X (mid-year estimate of the resident population). |
| **2. Number of persons with type 1 diabetes (all ages) in year X.**  Number of patients (unique person identifier) with primary healthcare unit/centre visit or hospital discharges and any listed diagnosis code for diabetes type 1 (ICD-10-WHO E100-E109) or registry patient.  *Exclusion criteria:* other types of diabetes E110-E149, transfers from a different hospital, non-resident patients, cases with MDC 14 or pregnancy, childbirth and puerperium codes in any field (ICD-10-WHO A34, F530-F539, O00-O99, Z320-Z392, Z640). All persons deceased till X-1 year need to be excluded. |  |
| **3. Number of persons with type 2 diabetes (all ages) in year X.**  Number of patients (unique person identifier) with primary healthcare unit/centre visit or hospital discharges and any listed diagnosis code for diabetes type 2 (ICD-10-WHO E110-E119) or registry patient  *Exclusion criteria:* other types of diabetes E100- E109, E120-E149, transfers from a different hospital, non-resident patients, cases with MDC 14 or pregnancy, childbirth and puerperium codes in any field (ICD-10-WHO A34, F530-F539, O00-O99, Z320-Z392, Z640). All persons deceased till X-1 year need to be excluded. |  |

## CHIEF-diabetes Indicator 3: Complications at diagnosis – retinopathy prevalence rate (%)

| **Numerator** | **Denominator** |
| --- | --- |
| **1. Number of persons with diabetic retinopathy at diagnosis (all ages).**  Number of patients (unique person identifier) with primary healthcare unit/centre visit or hospital discharges and any listed diagnosis code for diabetes (ICD-10-WHO E100-E119, E130-E149) and diabetic retinopathy (ICD-10-WHO H360) or registry patient with diabetic retinopathy (ICD-10-WHO H360) that were not diagnosed previously.  *Exclusion criteria:* transfers from a different hospital, non-resident patients, cases with MDC 14 or pregnancy, childbirth and puerperium codes in any field (ICD-10-WHO A34, F530-F539, O00-O99, Z320-Z392, Z640). | Total number of incident persons with diabetes in country in year X as end-year estimate of the resident population with incident diabetes, i.e. persons with listed diagnosis code for diabetes (ICD-10-WHO E100-E119, E130-E149 or persons from disease registry that were not diagnosed previously. |

## CHIEF-diabetes Indicator 4: Major lower extremity amputation incidence rate (per 1000 persons with diabetes annually, aged ≥15 years)

| **Numerator** | **Denominator** |
| --- | --- |
| **Number of persons with diabetes aged ≥15 years with major lower extremity amputation in year X.**  Number of patients (unique person identifier) with hospital discharges and any listed diagnosis code for diabetes (ICD-10-WHO E100-E119, E130-E149) and any listed procedure code for lower-extremity amputation (ACHI codes 44367-00[1484], 44370-00[1484], 44373-00[1484], 44367-01[1505], 44367-02[1505], 44361-00[1533], 44361-01[1533]) in a specified year  *Exclusion criteria:* Ages <15 years, transfers from a different hospital, non-resident patients, cases with MDC 14 or pregnancy, childbirth and puerperium codes in any field (ICD-10-WHO A34, F530-F539, O00-O99, Z320-Z392, Z640), cases with any listed diagnosis code for traumatic amputation (ICD-10-WHO S780–S789, S880–S889, S980–S984, T053–T055, T136), cases with any listed diagnosis code for tumours-related peripheral amputation (ICD-10-WHO C402–C403), lengths of stay <1 day (same-day admissions), malnutrition-related diabetes and toe amputations | Total number of persons with diabetes aged ≥15 years in country in year X (end-year estimate of the resident population aged ≥15 with diabetes, i.e. persons with listed diagnosis code for diabetes [ICD-10-WHO E100-E119, E130-E149] or persons from disease registry). |

## CHIEF-diabetes Indicator 5: Blindness rate (per 1000 persons with diabetes)

| **Numerator** | **Denominator** |
| --- | --- |
| Number of patients (unique person identifier) with primary healthcare unit/centre visit or hospital discharges and any listed diagnosis code for diabetes (ICD-10-WHO E100-E119, E130-E149) and blindness or low vision (ICD-10-WHO H540-H547) or registry patient with blindness or low vision (ICD-10-WHO H540-H547).  *Exclusion criteria:* transfers from a different hospital, non-resident patients, cases with MDC 14 or pregnancy, childbirth and puerperium codes in any field (ICD-10-WHO A34, F530-F539, O00-O99, Z320-Z392, Z640). All persons deceased up until year X-1 need to be excluded. | Total number of persons with diabetes in country in year X (end-year estimate of the resident population with diabetes, i.e. persons with listed diagnosis code for diabetes [ICD-10-WHO E100-E119, E130-E149] or persons from disease registry). |

## CHIEF-diabetes Indicator 6: Chronic renal failure rate – end stage renal disease (ESRD) (per 1000 persons with diabetes)

| **Numerator** | **Denominator** |
| --- | --- |
| Number of patients (unique person identifier) with primary healthcare unit/centre visit or hospital discharges and any listed diagnosis code for diabetes (ICD-10-WHO E100-E119, E130-E149) and ESRF (ICD-10-WHO N180-N186, Y841, Z49.1) or registry patient with ESFR (ICD-10-WHO N180-N186, Y841, Z49.1).  *Exclusion criteria:* transfers from a different hospital, non-resident patients, cases with MDC 14 or pregnancy, childbirth and puerperium codes in any field (ICD-10-WHO A34, F530-F539, O00-O99, Z320-Z392, Z640). All persons deceased up until year X-1 year need to be excluded. | Total number of persons with diabetes in country in year X (end-year estimate of the resident population with diabetes, i.e. persons with listed diagnosis code for diabetes [ICD-10-WHO E100-E119, E130-E149] or persons from disease registry). |
